# Species-specific mechanisms of tumor suppression are fundamental drivers of vertebrate speciation: critical implications for the ‘war on cancer’

**DOI:** 10.1530/ERC-18-0468

**Published:** 2018-10-12

**Authors:** Jonathan W Nyce

**Affiliations:** ACGT Biotechnology, Collegeville, Pennsylvania, USA

**Keywords:** p53, TP53, tumor suppression, war on cancer, mouse models, cancer

## Abstract

We recently reported our detection of an anthropoid primate-specific, ‘kill switch’ tumor suppression system that reached its greatest expression in humans, but that is fully functional only during the first twenty-five years of life, corresponding to the primitive human lifespan that has characterized the majority of our species' existence. This tumor suppression system is based upon the kill switch being triggered in cells in which p53 has been inactivated; such kill switch consisting of a rapid, catastrophic increase in ROS caused by the induction of irreversible uncompetitive inhibition of glucose-6- phosphate dehydrogenase (G6PD), which requires high concentrations of both inhibitor (DHEA) and G6P substrate. While high concentrations of intracellular DHEA are readily available in primates from the importation and subsequent de-sulfation of circulating DHEAS into p53-affected cells, both an anthropoid primate-specific sequence motif (GAAT) in the glucose-6-phosphatase (G6PC) promoter, and primate-specific inactivation of *de novo* synthesis of vitamin C by deletion of gulonolactone oxidase (GLO) were required to enable accumulation of G6P to levels sufficient to enable irreversible uncompetitive inhibition of G6PD. Malignant transformation acts as a counterforce opposing vertebrate speciation, particularly increases in body size and lifespan that enable optimized exploitation of particular niches. Unique mechanisms of tumor suppression that evolved to enable niche exploitation distinguish vertebrate species, and prevent one vertebrate species from serving as a valid model system for another. This here-to-fore unrecognized element of speciation undermines decades of cancer research data, using murine species, which presumed universal mechanisms of tumor suppression, independent of species. Despite this setback, the potential for pharmacological reconstitution of the kill switch tumor suppression system that distinguishes our species suggests that ‘normalization’ of human cancer risk, from its current 40% to the 4% of virtually all other large, long-lived species, represents a realistic near-term goal.

## Introduction

Ever since Darwin, biologists have been unsuccessful at achieving a consensus regarding a description of species, and the process of speciation (Mayden 1997, Hey 2006). Unlike Darwin, however, modern workers have had knowledge of the fundamental biology and physics of DNA for more than 50 years (Foulkes *et al*. 2018, Sussman 2018). At first glance, it may thus seem surprising that descriptions of species and the process of speciation have eluded us for so long. However, proper descriptions of species and speciation may not have been possible until the recent discoveries of species-specific mechanisms of tumor suppression, which we recently described in our Review paper ‘Detection of a primate-specific ‘kill switch’ tumor suppression mechanism that may fundamentally control cancer risk in humans’ (Nyce 2018), published in the November 2018 issue of *Endocrine-Related Cancer*. As we define in some detail in Supplementary Section 1 (see section on supplementary data given at the end of this article), the insight provided by these new discoveries indicate that malignant neoplastic transformation represents a fundamental counterforce to successful vertebrate speciation; that it originated as such a counterforce as a consequence of the evolution of closed circulatory systems in vertebrate animals; and that in response to this new counterforce to speciation, species-specific mechanisms of tumor suppression necessarily evolved as a prerequisite for expansion of body size and lifespan beyond that of species basal to a lineage. Species-specific mechanisms of tumor suppression are thus fundamental elements of vertebrate speciation, and every vertebrate species has evolved its own, unique tumor suppression strategy to achieve the body plan that enables it to optimize resource assimilation. These unique tumor suppression strategies fundamentally distinguish vertebrate species.

How is this important to the field of human cancer? The answer to this question is sobering. There is not a single drug in use in cancer today that did not fundamentally depend upon the use of murine species in its discovery and development, and the use of such model species is predicated upon there being a universal mechanism of action of p53, the ancient tumor suppression nexus from which cellular responses to potentially transforming DNA damage are distributed. However, as noted above, newly described species-specific mechanisms of tumor suppression fundamentally distinguish vertebrate species, such that one vertebrate species cannot be used to construct a valid model system describing cancer in another. There is not a single class of cancer drugs for which this predicament does not apply. This is exemplified by the fact that whereas p53 has long been called ‘the guardian of the genome,’ it is now increasingly also referred to as ‘the guardian of immune integrity,’ with its mutation-status having the ability to modulate the immune state of both its micro- (for example, Fig. 3 in Munoz-Fontela *et al.* 2016) and macro-environments (Pedersen *et al*. 2013).

## An apparent *lex naturalis* of vertebrate speciation

**Figure 1:**
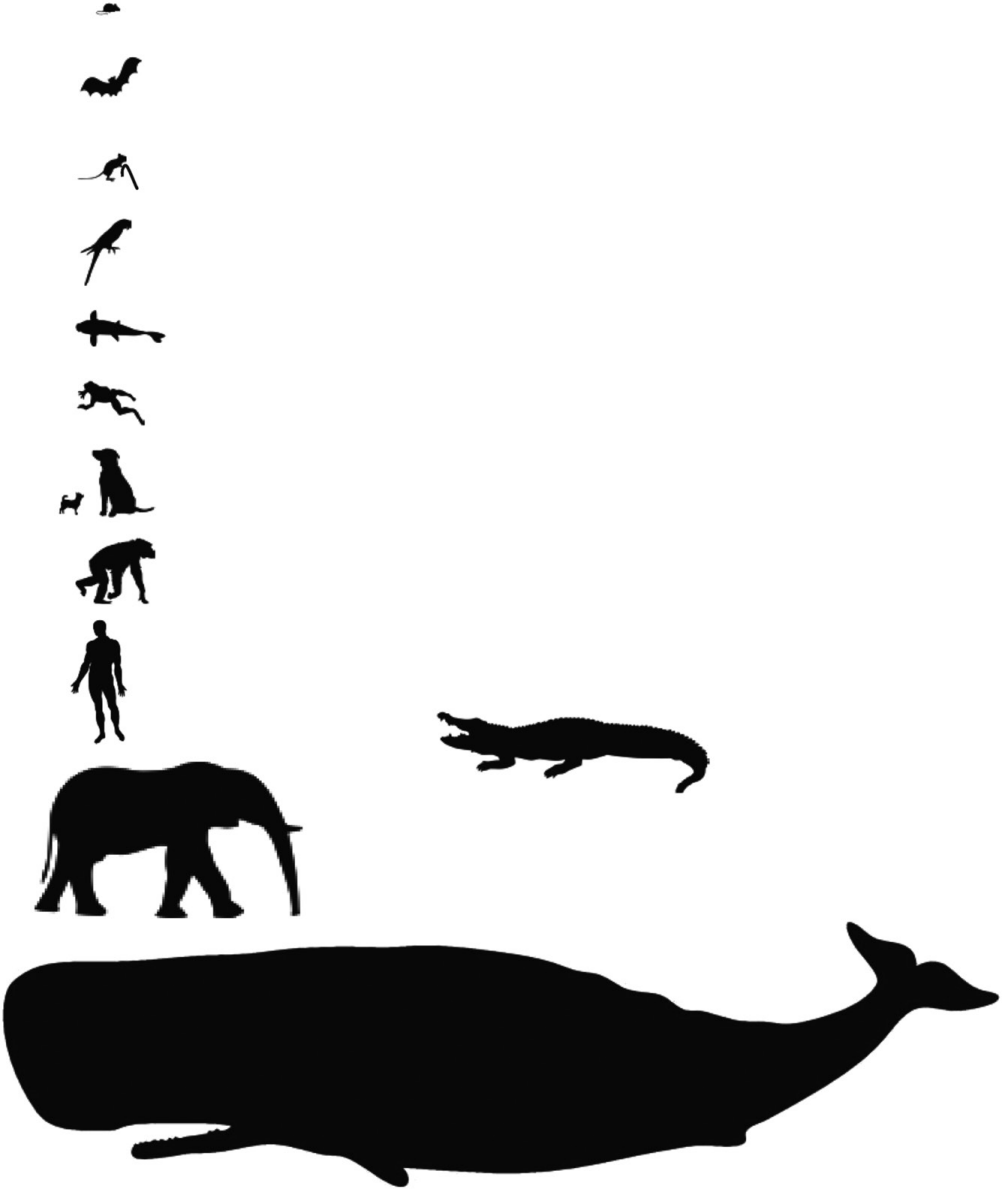
In vertebrate animals, species-specific mechanisms of tumor suppression enable species-specific adaptations such as body size and lifespan that, in turn, enable maximized exploitation of a species-specific niche. Thus, unique mechanisms of tumor suppression distinguish vertebrate species. So far as neoplastic transformation is concerned, species-specific mechanisms of tumor suppression prevent one vertebrate species from serving as a valid model system for another. This largely unrecognized element of speciation undermines decades of cancer research data, using murine species, which presumed universal mechanisms of tumor suppression, independent of species.

Because of their small size and short lifespan, mice evolved an economical, minimalist approach to tumor suppression in which the canonical p53 repertoire is sufficient to deal with cells that have suffered potentially transforming mutations. Humans, however, because of their dramatically increased body size compared to mice, had to evolve a totally different means of dealing with such good cells gone bad. The species-specific tumor suppression system of mice thus depends upon *activation* of p53 when DNA is damaged, resulting in the transcription of a cascade of DNA repair mechanisms; if the damage is too great, senescence or apoptosis is induced (the canonical p53 repertoire; Meek 2015). In stark contrast, the human-specific ‘kill switch’ tumor suppression system is triggered by the *inactivation* of p53, as described in Nyce (2018). Thus, a mutation in a p53 gene in a mouse tumor can reasonably be interpreted as a probable cause of the tumor, but a similar mutation in a human tumor represents something very different; *it is a fossil of kill switch tumor suppressor failure* – the failure of a system designed for the primitive human lifespan of approximately 25 years. Murine and human tumors are thus very different entities, literally as different as the species themselves. Consequently, for the past 40 years and more, we have been generating species-specific cancer data for species other than our own, and our efforts have unwittingly created drugs designed to treat cancer in mice, not humans; the extent to which such cancer drugs also work in humans is thus likely to be accidental. This is clearly reflected in the disappointing 7% improvement in 2-year survival observed in cancer patients over the past 27 years (https://surveillance.cancer.gov/statistics/types/survival.html; and Fig. 6 in Nyce (2018)). Would we have had better results if we had used an anthropoid primate, for example a Rhesus macaque, as our experimental model of human cancer for all these decades? Probably not. A macaque has only about one tenth the body mass of a human, and therefore, did not require full expression of the anthropoid primate-specific tumor suppression system that reached full expression only in humans, as discussed in Nyce (2018) and in Supplementary Section 2. Thus, to the extent that every vertebrate species has a unique body plan and lifespan, and has evolved unique methods to optimize its ability to assimilate environmental resources into DNA, the tumor suppression system of every vertebrate animal will be unique to its species. Therefore, again, one vertebrate species cannot be used to construct valid model systems of cancer for another vertebrate species (Fig. 1).

## The metastability of DNA drives speciation

The discovery of species-specific mechanisms of tumor suppression encourages a reductionist contemporary view of the driving force of speciation. Thus, as discussed in Supplementary Section 1, it is the entropy inherent in the mutability of DNA that drives speciation forward, with mutations that turn out to be ‘adaptive’ being those that enable DNA to create more of itself. More DNA equates to a greater number of spontaneous mutations due to the metastable nature of DNA, increasing disorder within the system, i.e., entropy constantly increases as the amount of DNA increases, driving the process of speciation forward. According to this contemporary view, species have been selected as the basic machines of evolution because they represent efficient mechanisms for the assimilation of environmental resources into DNA. But it is the entropy inherent in the mutability of the collective mass of DNA on this planet that drives speciation forward. Malignant transformation too, is driven forward by the entropy inherent in the mutability of DNA. But whereas speciation is the assimilation of environmental resources into information, malignant transformation is the assimilation of environmental resources into disinformation; i.e., malignant transformation fundamentally opposes successful vertebrate speciation, and this fact underlies the evolution of species-specific mechanisms of tumor suppression. These concepts are developed more fully in Supplementary Section 1.

## Speciation is a process that occurs in spacetime

The contemporary description of speciation encouraged by the discovery of species-specific mechanisms of tumor suppression also reveals that speciation is a process that occurs in space-time. This should not be too surprising, because biology occurs within the same physical framework that physicists have been describing for many decades. Physicists from Albert Einstein to Max Tegmark have tried to teach that, in a very fundamental sense, the past, the present and the future exist simultaneously. Thus, while time may have a directional arrow for all practical purposes, pointing in the direction of increased entropy, it does not have a precise ‘now’. Tegmark has described it this way: ‘There is nothing in the laws of physics that picks out one “now” over any other “now”’. It is just from our subjective viewpoint that it feels like things are changing’. As we describe in Supplementary Section 1, the process of speciation, similarly, does not have a precise ‘now’. This is particularly evident in the evolutionary strategies developed by small, short-lived animals to both optimize assimilation of environmental resources into DNA and to mitigate neoplastic transformation as an opposing force to such successful assimilation.

## A contemporary description of species and speciation

The absence of an inclusive description of species and the process of speciation that recognized species-specific mechanisms of tumor suppression as a fundamental element of vertebrate speciation was clearly permissive for the establishment and longevity of the p53 paradigm. Based upon insights gained by the recognition of such species-specific mechanisms of tumor suppression, Supplementary Section 1 also provides a contemporary description of species and the process of speciation. Without such an improved description, it would be difficult to unseat the current p53 paradigm. If we do not abandon this paradigm, we will continue to generate species-specific cancer data, and cancer drugs, for species other than our own.

## GLO deletion optimized kill switch function

In this commentary, we wish also to draw the reader’s attention to Supplementary Section 2 ‘Vitamin C auxotrophy is a vital component of the anthropoid primate-specific “kill switch” tumor suppression system.’ Thus, in addition to the anthropoid-primate-specific components of the kill switch described in Fig. 2 of Nyce (2018), in Supplementary Section 2, we now add the loss of the ability to synthesize ascorbate (by deletion of gulonolactone oxidase (GLO)) to that list and show that such loss was also necessary to bring the kill switch tumor suppression system into existence because GLO activity would act as a major sink for G6P.

It cannot be coincidental that three major, but here-to-fore unexplained, primate-specific evolutionary events – high levels of circulating DHEAS, the GAAT sequence motif of G6PC, and inactivation of the ability to synthesize vitamin C by deletion of GLO – have a common intersection in the enabling of irreversible uncompetitive inhibition of G6PD. Their juncture at uncompetitive inhibition of G6PD constitutes substantial evidence for the stepwise evolution of the anthropoid primate-specific kill switch tumor suppression system, culminating in humans (Supplementary Section 2).

## Is kill switch failure pharmacologically reversible?

However, such culmination of the kill switch in humans evolved with respect to the 25-year lifespans of primitive members of our species and is dysfunctional in modern humans as a result of a dramatic age-associated decline in circulating DHEAS. But unlike the kill switch mechanisms of other long-lived species, such as the elephant, which are genetic, the kill switch mechanism of humans is based upon a small molecule, DHEAS, and is therefore pharmacologically tractable. For the first time in the history of our species, a clear path forward toward the control of cancer may therefore have emerged. While cure may never be possible due to the virtually infinite heterogeneity of established tumors, identification of the human kill switch tumor suppression mechanism may enable ‘normalization’ of human lifetime cancer risk from its current 40%, to the 4% of other long-lived species such as the elephant. In our view, such normalization of cancer is a much more realistic goal than cure and would relegate cancer to a relatively rare disease in our species, as it is in most others.

Determining if reconstitution of circulating DHEAS will restore the kill switch tumor suppression system – and if such restoration will normalize human lifetime cancer risk to the 4% of other long-lived vertebrate species – is an experiment that can only be performed in humans. But unlike DHEA, DHEAS should be a safe pharmacological test substance. In view of this fact, and the dramatic increases in worldwide cancer cases predicted for the near future, we recommend that consideration be given to including the cost of such clinical trials in the National Cancer Institute’s 2020 budget, which has just been opened for consideration.

## Supplementary Material

Supplementary Section 1

Supplementary Section 2

## Declaration of interest

Prof. Nyce is a listed author on patent applications relating to methods to pharmacologically maintain kill switch function throughout the modern human lifespan; patent applications which he is willing to transfer to the National Cancer Institute under certain conditions.

## Funding

This work was funded in part by the U.S. Food and Drug Administration (G003126-C-2195-SR1891; G003126-C-2195-SR1892; G003126-C-2195-SR1893; G003126-C-2195-SR1894; G003126-C-2195-SR1895 to J N).
