# Supplementary material for: Species-specific mechanisms of tumor suppression are fundamental drivers of vertebrate speciation: critical implications for the ‘war on cancer’
Source: Endocr Relat Cancer. 2018 Oct 12;26(2):C1–5. doi: 10.1530/ERC-18-0468 (PMC6347260; doi:10.1530/ERC-18-0468)
Supplement: Supplementary Section 1 [file supplementary_section_1.pdf]

Supplementary Section 1

Species-specific mechanisms of tumor suppression are fundamental drivers of vertebrate speciation: Critical implication for the *war on cancer*

S1.1 The discovery of species-specific mechanisms of tumor suppression encourages a reductionist contemporary view of speciation

Speciation is the process by which the resources of a closed system, such as the earth, are assimilated into DNA. Species have been selected as the basic machines of evolution because they are efficient mechanisms for the assimilation of environmental resources into DNA. However, it is the entropy inherent in the mutability of the collective mass of DNA on this planet (Σ_{DNA}), in the presence of a constant energy source (E_c), that drives the movement and expansion of species through spacetime, with environmental resources capable of being assimilated into DNA (and DNA accessory molecules) acting as attractors for such movement. The mutability of the code it carries thus enables DNA to respond to its environment, because the mutations that turn out to be “adaptive” are those that enable DNA to assimilate attractors into more of itself, expanding its range. More DNA equates to a greater number of spontaneous mutations due to the metastable nature of DNA, increasing disorder within the system; i.e., entropy constantly increases as the amount of DNA increases. Because DNA is a code-carrying molecule, speciation can be alternatively described as the process by which the resources of a closed system, such as the earth, are assimilated into information (i_{DNA}). The disorder introduced into the system by random mutations in DNA is always larger than the amount of information produced; the movement and expansion of species through spacetime therefore always proceeds from a condition of less entropy toward one of increased entropy:

$$E_c \xrightarrow{DNA} i_{DNA} \;,\; \Sigma_{DNA} >> i_{DNA}$$

Neoplastic transformation directly opposes successful speciation in vertebrate animals. In the same manner that speciation is the conversion of environmental resources into information, cancer is the conversion of environmental resources into disinformation--disinformation that destroys the fitness of the soma and thereby acts as a counterforce to successful speciation. Because of such direct opposition, vertebrate speciation is characterized by species-specific mechanisms of tumor suppression that are required to advance body size and lifespan beyond those of species basal to a lineage. Thus, unique mechanisms of tumor suppression *distinguish* vertebrate species, and in-so-far as malignant neoplastic transformation is concerned, the existence of such species-specific mechanisms of tumor suppression prevent one vertebrate species from serving as a valid model system for another. This largely unrecognized element of vertebrate speciation undermines decades of cancer research data, using murine species, that presumed universal mechanisms of tumor suppression, independent of species.

S1.2 Closed circulatory systems ushered in the dawn of neoplastic transformation as a counterforce opposing successful speciation

Early forms of life were not endowed with the means to perform significant work. That is, they were sessile or nearly so, depending primarily upon environmental forces such as currents of water or air for significant displacement of their mass over a distance. Their range of habitat exploitation was consequently limited to their local environment, or to areas made accessible by water or air currents. With the evolution of chloroplasts, plant life expanded its realm dramatically, particularly in terrestrial environments where absorption of sunlight was unimpeded by the water column. This flourishing growth of terrestrial flora created an attractor of unrivaled dimensions, but one that remained unavailable to aquatic species, the then dominant life forms. While certain species of

invertebrates evolved significant mass and were capable of displacing such mass over asignificant distance in aqueous environments (e.g., *Anomalocaris, Mesonychoteuthis*), it was the evolution of closed circulatory systems in vertebrate animals that enabled the exploitation of terrestrial flora. Thus, by dramatically increasing the ability of vertebrate animals to perform work, closed circulatory systems enabled some originally obligatorily water-borne species to access the attractor of terrestrial plant life. With subsequent refinement of such closed circulatory systems, particularly with respect to the evolution of hearts with improved pumping efficiency, vertebrates that adapted to the land expanded their ability to conduct locomotion over significant terrestrial distances, making virtually all terrestrial plant resources available for assimilation into DNA. Closed circulatory systems also enabled vertebrate animals to resist the force of gravity and extend their spectrum of possible body sizes across an incredible range. Without closed circulatory systems capable of highly oxygenating tissues, the evolution of bones, muscle, body mass and stature observed in vertebrate animals would not have been possible, leaving enormous concentrations of resources unavailable for assimilation.

The improvements in capacity to perform work and the potential to increase body size that were brought about by closed circulatory systems clearly increased the extent to which, and the rate at which, environmental resources could be assimilated into DNA. However, they also brought into prominence a counterforce to resource assimilation—neoplastic transformation— that had previously not had much impact on such resource assimilation. First, the dramatic increase in body size enabled by closed circulatory systems increased the risk of neoplastic transformation in large vertebrate animals due to an equivalently dramatic increase in the number of stem cells at risk for such neoplastic transformation. All things being equal, an organism with 10,000 times the mass of another would be expected to have 10,000 times the risk of neoplastic transformation.

However, risk of neoplastic transformation does not scale with body size (Peto's paradox), which can now be explained by the fact that species-specific mechanisms of tumor suppression are a necessary prerequisite for any significant increase in vertebrate body size above that of species basal to a lineage (see below). Without simultaneously evolving such species-specific mechanisms of tumor suppression, animals such as humans, elephants and whales could not attain size and lifespan so dramatically in excess of that of species basal to their respective lineages.

Second, the evolution of closed circulatory systems enabled neoplastic growths, for the first time, to achieve widespread dispersion throughout the soma of an organism, reducing fitness by negatively impacting the function of tissues and organs which they have invaded. In animals with open circulatory systems, such as arthropods, in which blood is pumped into spaces bathing tissues and organs rather than into the tissues and organs themselves, a direct conduit into tissues and organs is absent. Only in animals with closed circulatory systems does the vasculature provide a pressurized conduit to virtually every compartment of the soma. Spontaneous explants from primary neoplastic growths that enter such a closed circulation thus have access to all compartments of the soma—the process of metastasis. It is well known that in our own species it is generally not the primary neoplasm that diminishes fitness and kills the organism, but rather organ failure caused by invasion and metastases (Fidler and Kripke, 2015). Cancer is in fact defined as the ability of a neoplastic growth to invade and metastasize; neoplasms with this capacity are termed malignant, while neoplasms without this capacity are termed benign for their general failure to significantly reduce fitness of the organism. Furthermore, whereas tissues and organs develop as a result of the organized expression of genetic and epigenetic programs that specify appropriate blood supply, neither primary nor metastatic tumors, which operate with disregard for such programs, have natural blood supplies.

Rather, as they grow in the absence of proper oxygenation, they become hypoxic and activate angiogenesis, the attraction of new blood vessel growth into their mass (Folkman, 2006), drawing away from the soma the resources required to maintain the fitness of the organism. The ability of neoplastic growths to metastasize to distant sites and thereby destroy fitness of the organism is thus a direct result of the evolution of closed circulatory systems. Malignant neoplastic transformation is thus a fundamental force opposing successful speciation that must be countered by species-specific mechanisms of tumor suppression in vertebrate species, particularly in those whose exploitation of environmental niche is improved by increased body size and lifespan.

S1.3 The *min-max* equation of speciation in vertebrate animals

The process of speciation in vertebrate animals appears to be optimized when a certain background level of somatic cell genome instability, i.e., malignant neoplastic transformation, is permitted to occur. Thus, in a sort of *min-max* equation designed to create species capable of assimilating resources into DNA at the maximum possible rate, lifetime risk of neoplastic transformation is maintained at a low, but generally non-zero level. That is, a certain minimized but nonzero amount of neoplastic transformation occurs when the rate of assimilation of resources into DNA by a species is maximized. A recent large, multi-center effort studying cancer risk across a wide array of placental mammals put the background level of neoplastic transformation at about 4% (Abegglen *et al.*, 2015). Part of this background level of somatic genome instability may be a consequence of the sensitivity of nucleophilic centers in DNA to substitution reactions initiated by ubiquitous electrophiles, and part may be due to the evolution of DNA methylation as a means to selectively silence the transcription of specific genes during the development of complex vertebrate bodies (Nyce *et al*., 1983; Baylin and Jones, 2016), and also to control the activity of transposable elements in chromosomes (Ohtani *et al*., 2018). Enzymatic DNA

methylation produces 5-methylcytosine (5mC), and such 5mC moieties are well-known to be hyper-mutable via spontaneous deamination, producing thymine (Ehrlich *et al*., 1986; Poulos *et al*., 2017). Since successful vertebrate species appear to be characterized by a low but non-zero level of neoplastic transformation in their soma, it is possible that this represents an irreducible value inherent in the metastability of DNA and the hyper-mutability of 5mC. However, some vertebrates appear to have lifetime risks of cancer that approach zero in their natural, species-specific habitat (Azpurua and Selanov, 2012; Delaney *et al.*, 2013), despite the fact that they use the same metastable DNA and hyper-mutable 5mC as other vertebrate species. It would thus appear that the maintenance of virtually pristine somatic genomes is possible and could be accomplished if that was the ultimate goal of speciation. However, it is apparently *not* the ultimate goal; rather, maximizing the assimilation of environmental resources into DNA appears to be the ultimate goal of speciation, and assimilation of environmental resources into DNA appears to be maximized when a certain minimum, but non-zero level of neoplastic transformation is permitted to occur in the soma of vertebrate animals.

S1.4 The apparent *lex naturalis* of vertebrate speciation: Body size, lifespan, species-specific mechanisms of tumor suppression, and lifetime cancer risk are tightly connected elements of vertebrate speciation, such that alteration in one will necessarily produce an equilibrating change in one or more of the others

If an equation describing a vertebrate species could be written, and if variables in that equation associated with the suppression of malignant neoplastic transformation could be manipulated, then either increasing *or* decreasing such a variable would be counteracted by an equilibrating change in another of the above-cited variables, preventing a decrease in the rate of resource assimilation into DNA. An experiment along these lines has already occurred by the manipulation of the size of various dog breeds by humans. In large dog

breeds, such as the Great Dane, and the Bernese Mountain Dog, the artificially induced increase in the variable of body size (above that naturally attained by the wolf) has resulted in an equilibrating decrease in the variable of lifespan. Similarly, by artificially decreasing body size in other breeds of dogs, such as the Chihuahua and the Pomeranian, the variable of lifespan has been correspondingly increased. If Great Danes, Bernese Mountain Dogs, Chihuahuas and Pomeranians had evolved naturally, without human intervention, they would have become discrete species, with different application of canine lineage-specific tumor suppression mechanisms, similar to the manner in which different anthropoid primate species deployed the kill switch tumor suppression system to differing extents depending upon their needs (Supplementary Section 2). However, humans effected the increase in size in Great Danes and Bernese Mountain dogs without providing them with an improved tumor suppression strategy the way nature would have done to offset the increased risk of neoplastic transformation associated with such increased size. This action of artificial selection for large body size thus resulted in an opposite and equal reaction, producing a decrease in lifespan in these large breed dogs. Small dogs, on the other hand, released from some of the more stringent pressures of natural selection because they were under the protection of humans, enjoyed the benefits of small size, in which the canonical p53 repertoire is better able to protect them than it is in larger dogs (Nyce, 2018; and section S1.5 below). Although intensive in-breeding has obscured comparisons of lifetime cancer risk in dogs, the consistent fact emerges that the smallest canine breeds (e.g., Chihuahuas, Pomeranians, Miniature Pinschers) have significantly longer lifespans (Patronek *et al*., 1997; Michell, 1999), in accordance with the apparent *lex naturalis* of vertebrate speciation: *Body size, lifespan, species-specific mechanism of tumor suppression, and lifetime cancer risk are tightly connected elements of speciation, such that alteration in one will necessarily produce an equilibrating change in one or more of the others.*

In modern humans, lifespan and body size have both been increased by means (modern medicine, the modern economy) that divorce our species from its speciation history, with increased longevity disabling our species-specific tumor suppression mechanism which evolved for the 25 year lifespans of primitive humans (Nyce, 2018). Still, the *lex naturalis* holds: Since the modern human lifestyle disables equilibration between the variables of lifespan, body size, and tumor suppression mechanism, equilibrium is unfortunately restored by an increase in lifetime cancer risk. However, with the discovery of the kill switch tumor suppression system, and the apparent capacity to reconstitute it pharmacologically, it may now be possible to balance the equation for our species such that lifetime cancer risk is restored to the normal level of 4% for vertebrate animals. We might also learn from our manipulation of canine stature that short stature should replace tall as the ideal human form. Our continued preference for tall stature is a relic of its value in a primitive landscape in which the ability to hunt with primitive weapons and perform other strength-intensive tasks, and in which predation by large carnivores, and inter-tribal warfare, applied strong selective pressures that are no longer relevant in the modern world. In as much as height has been conclusively demonstrated to be directly related to cancer risk in humans (Green et al., 2011; Kabat et al., 2013; Ong et al., 2018), a significant reduction in average stature would accrue many advantages to future human societies, not least of which would be a decrease in lifetime cancer risk as we experience further increases in lifespan.

S1.5 Speciation is a process occurring in spacetime, without a specific "now"

Speciation strategies are executed in spacetime; that is, the accounting period for optimization of resource assimilation into DNA, as well as the debiting of the negative impact of neoplastic transformation, is not limited to the present, or even to the lifespan of the vertebrate animal under consideration. In fact, as noted above, lifespan is a variable that species can manipulate to optimize their ability to assimilate environmental resources

into DNA. Thus, the accounting period for net assimilation of resources into DNA can include many generations, particularly for small, short-lived vertebrate animals. Species are thus collections of animals dispersed not only over space, but also over time, such that the representation of a species on a spacetime curve would show a roughly spherical mass extended not only into the past, but also into the future. The time dimension of such spherical masses can be thought of as species-specific quanta which incorporate the timescale used by different species in their strategies to maximize assimilation of environmental resources into DNA, while simultaneously minimizing opposing forces such as malignant neoplastic transformation. For smaller, short-lived animals, their quanta appear to encompass a span of multiple generations. For example, mice and elephants execute very different strategies of resource assimilation and mitigation of neoplastic transformation, but over the long term, for example the lifetime of an elephant (which encompasses many generations of mice), one female animal of each species can initiate the production of roughly equivalent amounts of DNA assimilated from environmental resources (after accounting for significant predation of the mouse population, mice representing an environmental resource for many other species). The flexibility of the quantum of time over which speciation may operate appears to bear particularly upon the execution of strategies to mitigate malignant transformation in vertebrate animals. Thus, small body size and short lifespan are frequently employed evolutionary strategies of basal species. This strategy has been so successful that it continues in extant species. For example, small body size in mice enables both their commensal existence with humans and limits the number of stem cells at risk for neoplastic transformation. Utilizing the time dimension of their spacetime environment to further minimize cancer risk, the short lifespan of mice resets accumulated mutations in somatic cells to near zero at frequent intervals in successive generations, spreading risk of neoplastic transformation across time. Small body size coupled with short lifespan can thus be considered an extremely

effective means of tumor suppression, requiring only the canonical repertoire of p53 to maintain a low cancer risk and achieve an optimized rate of resource assimilation into DNA. A recent study of inbred mice (which likely have a substantially higher incidence of spontaneous cancer than outbred mice in the wild) demonstrated that the incidence of tumors was approximately 11 per 5,000 animals per year, representing a lifetime risk of 0.22-0.44% (Marx *et al*., 2013). These data support the concept that if small, short-lived animals such as mice hold true to the 4% cancer risk as observed in other vertebrate species (Abegglen *et al*., 2015), then this risk in small, short-lived animals is not based upon the body mass of a single individual, but rather on the body mass represented by a collection of individuals; that is, a “collective body mass” spread across generations, across time.

S1.6 Evolution of body size and lifespan beyond that of basal species

Enlargement of body size and lifespan beyond that of species basal to a lineage in order to expand the range of assimilable resources may have been an early driver of species-specific mechanisms of tumor suppression. For example, the environmental resources that acted as attractors for elephant evolution, and for whale evolution, were best exploited by creatures with large body mass, which only becomes efficient with increased lifespan. To enable such increase in body mass and lifespan, elephants evolved expansion of their p53 compartment from the two alleles present in all animal species, adding to their genomes nineteen p53 retropseudogenes that retain tumor suppressor activity (Abegglen *et al., ibid*.; Sulak *et al*., *ibid*.). Thus, the canonical p53 repertoire so well studied utilizing the p53 knockout mouse is sufficient for mice with their small body size and short lifespan, but had to be augmented by the species-specific addition of p53 retropseudogenes in elephants which evolved large body mass and long lifespan to maximally exploit their environmental niche. Similarly, anthropoid primates, particularly humans with their species-specific hydrocarbon exposure (Supplementary Section 1 in Nyce 2018; and Supplementary

Section 2 in the present Commentary), also found it necessary to augment the canonical p53 repertoire with a lineage-specific tumor suppression system, at least during their adult phase. As noted above, height has been demonstrated to be directly related to cancer risk in humans (Green et al., 2011; Kabat et al., 2013; Ong et al., 2018). If this finding extrapolates across the entire range of human body sizes, the small bodies of human children, in combination with the canonical p53 repertoire, may be sufficient to maintain neoplastic transformation at the required low level at this stage of human development. However, with the transition to the much larger body size of adult humans, activation at adrenarche of the adrenal androgen-mediated kill switch tumor suppression system is required (Nyce, 2018). This human-specific kill switch tumor suppression system may have permitted primitive adult humans to retain the low cancer risk of their juvenile phase after transitioning to the increased stature of their adult phase.

The concept of species-specific mechanisms of tumor suppression economically employing the same mechanisms that enabled exploitation of a species' specific niche (Nyce, 2018) encourages interesting predictions. For example, deep diving cetaceans such as the sperm whale may utilize the elevated ROS caused by re-oxygenation after diving-induced hypoxia to selectively extinguish pre-malignant cells, doing so by withholding from such cells the enhanced anti-oxidant system that evolved to protect against re-perfusion injury in this species. Species-specific mechanisms of tumor suppression have already been reported in the bowhead whale, which is not only one of the most massive creatures currently alive, but also the longest-lived mammalian species (Keane *et al*., 2015). We further predict that the p53 retropseudogenes that appear to maintain lifetime cancer risk in the elephant to the requisite 4-5% will be found to participate also in the enablement of niche exploitation in this species.

The critical point to be made here is that, while the canonical repertoire of p53 may be sufficient to maintain risk of neoplastic transformation to the required low level in small,

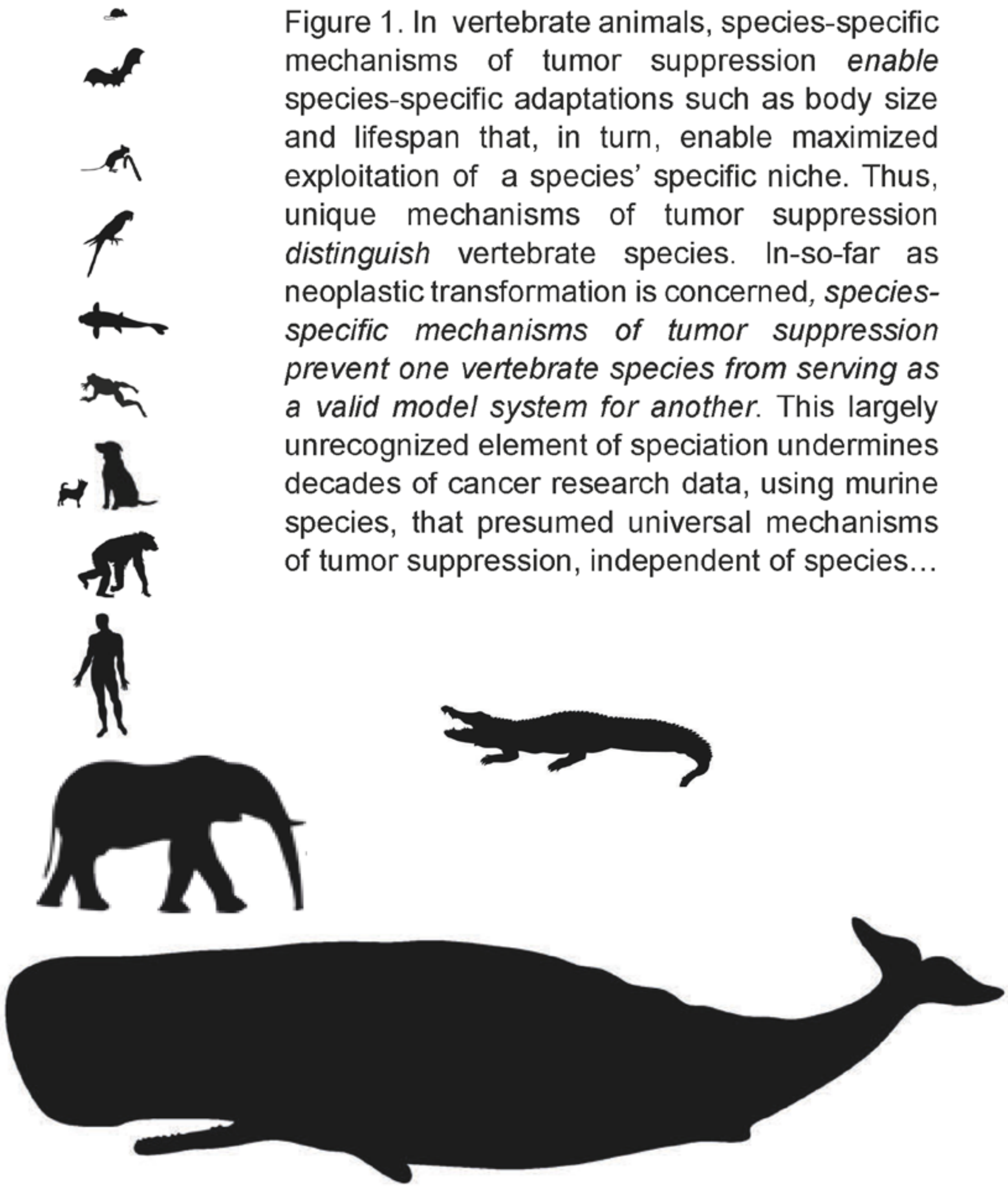

Figure 1. In vertebrate animals, species-specific mechanisms of tumor suppression *enable* species-specific adaptations such as body size and lifespan that, in turn, enable maximized exploitation of a species' specific niche. Thus, unique mechanisms of tumor suppression *distinguish* vertebrate species. In-so-far as neoplastic transformation is concerned*, species-specific mechanisms of tumor suppression prevent one vertebrate species from serving as a valid model system for another*. This largely unrecognized element of speciation undermines decades of cancer research data, using murine species, that presumed universal mechanisms of tumor suppression, independent of species…

short-lived animals (and in the juvenile phase of larger species), it is insufficient for this task in larger, longer-lived adult vertebrate animals. Such larger, longer-lived animals had to evolve additional species-specific mechanisms of tumor suppression that enabled their increased body mass and longevity (Figure 1). In direct opposition to the established p53 cancer paradigm, species-specific mechanisms of tumor suppression disqualify animals such as mice from acting as models for use in the study of human

cancer. The evolutionary spacetime trajectories of these species have been driven by completely different species-specific tumor suppression mechanisms. The virtually exclusive use of murine model systems may thus account for the disappointing 7% increase in two-year cancer survival that has been achieved over the past 27 years (U.S. NCI SEER Statistics, May 2018; Figure 5 in Nyce, 2018).

S1.7 The epigenome in vertebrate speciation and malignancy

The epigenome is a dynamic pattern of covalent and noncovalent imprints laid down upon DNA to control temporal and spatial aspects of gene regulation, enabling the development of complex body plans and even behaviors (McGowan and Roth, 2015). The epigenome has recently been demonstrated to act as a sensor of changing environmental conditions, able to prepare offspring to withstand such changes via adjustment of gene regulation-- on a time scale dramatically shorter than that which could occur by genetic mechanisms of natural selection (Rechavi *et al*., 2014). We have suggested that, in species with minimal genetic differences, the epigenome may direct divergence in body plan and method of niche exploitation; that changes in the epigenome may precede changes at the level of the genome during speciation; and that species-specific levels of DNA methylation and demethylation (covalent imprints) may also regulate Σ_{DNA} in a species-specific manner. With regard to malignant somatic cells, the ability of their epigenomes to respond to treatment challenge demonstrates the plasticity that has made cancer incurable up until now. As an example, DNA hypermethylation induced by exposure to certain cancer drugs can transcriptionally inactivate genes the products of which are required for drug activation, creating drug-resistant "epimutants" within tumor cell populations; under continued selection pressure, such "epimutants" can undergo mutation to permanently silence the epi-targeted gene (Nyce, 1997;1989; Nyce *et al*.,1993).

S1.8 A contemporary description of species and the process of speciation must include the contribution of species-specific mechanisms of tumor suppression

The absence of adequate descriptions of species and the process of speciation was clearly permissive for the establishment and longevity of the p53 tumor suppressor paradigm, and for the delusive animal models that this paradigm continues to support to the present day.

Without the recent insight gained from comparative studies, particularly that all vertebrate animal species evolved species-specific tumor suppression strategies that enable each species to traverse a unique vector through spacetime, adequate descriptions may have here-to-fore been impossible. A contemporary description of species and the process of speciation is now possible, and critically important if the established p53 paradigm is to be unseated and replaced with a more productive one.

Using insights gained from species-specific mechanisms of tumor suppression cited above, we here present a contemporary, inclusive description of species and the process of speciation.

1. Speciation is the process by which the resources of a closed system, such as the earth, are assimilated into DNA.
2. Species have been selected as the basic machines of evolution because they are efficient mechanisms for the assimilation of environmental resources into DNA; however, it is the entropy inherent in the mutability of the collective mass of DNA on this planet (Σ_{DNA}), in the presence of E_c, that drives speciation forward.
3. Environmental resources capable of being assimilated into DNA (and DNA accessory molecules) act as attractors for such forward movement.
4. DNA is an informational molecule. Therefore, speciation is the process by which the resources of a closed system, such as the earth, are assimilated into information ($\boldsymbol{i}_{DNA}$). Expansion of the collective mass of DNA always proceeds from a condition of lesser to one of greater entropy because the disorder introduced into the system by random mutations is always greater than the amount of information assimilated, i.e., $\Sigma_{DNA} >> \boldsymbol{i}_{DNA}$
5. Malignant neoplastic transformation can also be understood in these terms, as *disinformation* that *obstructs* the process of speciation, hence requiring its maintenance within controlled limits. Malignant neoplasia/tumor progression is also driven forward through spacetime by the entropy inherent in DNA mutation, but since disinformation is the product, $\Sigma_{malignant\ DNA} >>> \boldsymbol{i}_{DNA}$

6. All enabling elements of a species' forward movement through spacetime, including countermeasures taken against opposing forces such as neoplastic transformation, represent variables that are under combined selection to maximize assimilation of environmental resources into DNA.
7. In vertebrates, increase in body size and lifespan over that of species basal to a lineage is a common mechanism to expand niche exploitation beyond that of the basal species.
8. The evolution of closed circulatory systems enabled increased capacity for work (locomotion) and increased body size, factors capable of significantly enhancing the ability to assimilate attractors into DNA. However, closed circulatory systems also promoted neoplastic transformation to a process capable of reducing fitness in vertebrate animals by enabling metastatic spread. To counteract malignant neoplastic transformation as an opposing force to successful speciation, species-specific mechanisms of tumor suppression became necessary prerequisites to any increase in vertebrate body size and lifespan beyond that of basal species.
9. Based on data from extant mammalian species, maximum rate of assimilation of environmental resources into DNA by such species is achieved when lifetime risk of neoplastic transformation is maintained within limits approximating 4%.
10. Selection for biochemical economy frequently results in the same mechanism being used to both advance niche exploitation and maintain lifetime cancer risk at the requisite 4%.
11. Body size, lifespan, species-specific mechanisms of tumor suppression, and lifetime cancer risk are tightly connected elements of vertebrate speciation, such that alteration in one will necessarily produce an equilibrating change in one or more of the others.
12. Speciation is a process occurring within spacetime, such that the accounting period for optimization of resource assimilation into DNA, as well as the debiting of the negative impact of neoplastic transformation, can be spread over generations. Thus, in small, short-lived species such as mice, short lifespan resets accumulated mutations to near zero at very short intervals in successive generations, spreading risk of neoplastic transformation across time.

13. The original shape of a species as it moves along its trajectory through spacetime is roughly spherical, with a genetic center of mass; however, its shape can become amorphous as it comes into contact with new attractors, and regions of its shape (neomorphs) are driven toward such attractors by the entropy of DNA mutation. If a solution is found enabling assimilation of the new attractor, the budding off of a new species returns the progenitor species to a roughly spherical shape until contact with the next new attractor occurs.

14. The evolution of unique epigenomes also contributes to speciation. Unfortunately, at the level of somatic cells, it also contributes significantly to the plasticity of tumor cell response to all known treatment modalities.

15. A species is thus defined as a collection of similar genomes/epigenomes moving along the same spacetime trajectory toward the same attractor(s).

16. Biodiversity is the natural result of such entropy-driven movement through spacetime toward such attractors.

S 1.9 Conclusion

Since the War on Cancer was declared in the United States by President Nixon in 1971, more than 300 million Americans have been diagnosed with cancer-- which is roughly equivalent to the entire U.S. population today. The majority of these 300 million people died of their disease. Similar statistics exist throughout Europe and many parts of Asia. Both the National Cancer Institute and the World Health Organization have forecast that the number of patients with new diagnoses of cancer worldwide will reach 24 million *annually* by 2030. Clearly, our efforts up to now have not been successful at significantly stemming the impact that cancer has had and will continue to have on our species.

The p53 tumor suppressor paradigm as delineated in murine model systems represents the foundation upon which the discovery and development of all drugs for

human cancer, from the cytotoxic small molecule drugs to the newer, antibody-based immune checkpoint inhibitors, have so far been based. The discovery that malignant neoplasia is a fundamental force opposing speciation in vertebrate animals, and that species-specific mechanisms of tumor suppression have evolved as a counterforce enabling expansion of body size and lifespan beyond that of species basal to a lineage, provides clear evidence that murine species cannot be used to construct valid models of human cancer. Use of such models for the study of human cancer must end.

It may be impossible to “cure” developed tumors because of the incredible depth of their genetic heterogeneity and epigenetic plasticity. While the anthropoid primate-specific kill switch tumor suppression system evolved to protect during the 25-year lifespan of primitive humans, it shows the potential to be pharmacologically reconstituted to maintain kill switch function throughout the protracted lifespan of modern humans. This presents the realistic near term goal of "normalizing" human lifetime cancer risk from its current, abnormal 40%, to the 4% of virtually all other larger, long-lived species. In the face of the human suffering caused by cancer, the surety of its continuing advance in our species, and the unsustainable costs of new cancer medicines that only minimally extend life in cancer patients, such normalization may be preferable to a cure.

References

Abegglen LM, Caulin AF, Chan A, Lee K, Robinson R, Campbell MS, Kiso WK, Schmitt DL, Waddell PJ, Bhaskara S, Jensen ST, Maley CC, Schiffman JD 2015 Potential mechanisms for cancer resistance in elephants and comparative cellular repsonse to DNA damage in humans. *JAMA* **314**(17)1850-1860. (https://jamanetwork.com/journals/jama/fullarticle/2456041)

Azpurua J, Selanov A 2012 Long-lived cancer-resistant rodents as new model species for cancer research. *Front Genet* **3** 319-321. (https://www.ncbi.nlm.nih.gov/pmc/articles/PMC3540411/)

Baylin SB, Jones PA 2016 Epigenetic determinants of cancer. Cold Spring Harbor *Perspect Biol* **8**(9) piia019595. (https://www.ncbi.nlm.nih.gov/

Campbell BC 2006 Adrenarche and the evolution of human life history. *American Journal of Human Biology* **18**(5):569-589. (DOI: 10.1002/ajhb.20528)

Delaney MA, Nagy L, Kinsel MJ, Treuting PM 2013 Spontaneous histological lesions of the adult naked mole rat (Heterocephalus glaber): a retrospective survey of lesions in a zoo population. *Vet Pathol* **50**(4):607-621. (https://www.ncbi.nlm.nih.gov/pubmed/23355517)

Ehrlich M, Norris KF, Wang RY, Kuo KC, Gehrke CW 1986 DNA cytosine methylation and heat-inducible deamination. *Biosci Rep* **6**(4):387-393. (https://www.ncbi.nlm.nih.gov/pubmed/3527293)

Farooqi NAI, Scotti M, Lew JM, Botteron KN, Karama S, McCracken JT, Nguyen TV 2018 Role of DHEA and cortisol in prefrontal-amygdalar development and working memory. *Psychoneuroendocrinology* **98**:86-94. (doi: 10.1016/j.psyneuen.2018.08.010)

Fidler IJ, Kripke ML 2015 The challenge of targeting metastasis. *Cancer Metastasis Rev* **34**(4):635-641. (https://www.ncbi.nlm.nih.gov/pmc/articles/PMC4661188/)

Folkman J 2006 Angiogenesis. *Annu Rev Med* **57**:1-8. (https://doi.org/10.1146/annurev.med.57.121304.131306)

Geer KA, Canterberry SC, Murphy KE 2006 Statistical analysis regarding the effects of height and weight on life span of the domestic dog. Res Vet Sci 82(2):208-214. https://www.ncbi.nlm.nih.gov/pubmed/16919689

Green J, Cairns J, Casabonne D, Wright FL, Reeves G, Beral V 2011 Height and cancer incidence in the Million Women Study: prospective cohort, and meta-analysis of prospective studies of height and total cancer risk. *Lancet Oncology* **12**(8):785-794. (https://www.ncbi.nlm.nih.gov/pmc/articles/PMC3148429/)

Kabat GC, Anderson ML, Heo M, Hosgood HD, Kamensky V, Bea JW, Hou L, Lane DS, Wactawski-Wende J, Manson JE, et al. 2013 Adult stature and risk of cancer at different anatomic sites in a cohort of postmenopausal women. Cancer Epidemiology,

Biomarkers & Prevention **22**(8):1353-1363. (http://cebp.aacrjournals.org/content/22/8/1353)

Keane M, Semeiks J, Webb AE, Li Y, Quesada V, Craig T, Madsen LB, van Dam S, Brawand D, Marques PI, Michalak P, Kang L, Bhak J, Yim HS et al., 2015 Insights into the evolution of longevity from the bowhead whale genome. Cell Rep 10(1):112-122. Doi: 10.1016/j.celrep.2014.12.008

Marx JO, Brice AK, Boston RC, Smith AL 2013 Incidence rates of spontaneous disease in laboratory mice used at a large biomedical research institution. J Am Assoc Lab Anim Sci 52(6):782-791. (https://www.ncbi.nlm.nih.gov/pmc/articles/PMC3838613/)

McGowan P O, Roth T L 2015 Epigenetic pathways through which experiences linked with biology. Dev Psychopathol 27(2):637-648. https://www.ncbi.nlm.nih.gov/pmc/articles/PMC4489549/pdf/nihms-698503.pdf

Michell AR 1999 Longevityt of British breeds of dog and its relationship to size, cardiovascular variables and disease. Vet Rec 145(22):625-629. https://www.ncbi.nlm.nih.gov/pubmed/10619607

Nyce JW 2018 Detection of a novel, primate-specific 'kill switch' tumor suppression mechanism that may fundamentally control cancer risk in humans: an unexpected twist in the basic biology of p53. Endocrine-Related Cancer 25(11) R497-R517. https://doi.org/10.1530/ERC-18-0241

Nyce J 1997 Drug-induced DNA hypermethylation: a potential mediator of acquire drug resistance during cancer chemotherapy. Mutation Res 386(2):153-61. https://www.ncbi.nlm.nih.gov/pubmed/9113116

Nyce J, Leonard S, Canupp D, Schulz S, Wong S 1989 Epigenetic mechanisms of drug resistance: drug-induced DNA hypermethylation and drug resistance. Proc Natl Acad Sci USA 90(7):2960-4. https://www.ncbi.nlm.nih.gov/pmc/articles/PMC46216/

Nyce J 1989 Drug-induced DNA hypermethylation and drug resistance in human tumors. Cancer Res 49(21):5829-36. http://cancerres.aacrjournals.org/content/49/21/5829.long

Ohtani H, Liu M, Zhou W, Liang G, Jones PA 2018 Switching roles for DNA and histone methylation depend on evolutionary ages of human endogenous retroviruses. Genome Res 28(8):1147-1157. (https://www.ncbi.nlm.nih.gov/pubmed/29970451)

Ong J-S, An J, Law M, Whiteman DC, Neale RE, Gharahkhani P, MacGregor S, 2018 Height and overall cancer risk and mortality: evidence from a Mendelian randomization study on 310,000 UK Biobank participants. *British Journal of Cancer* **118**:1262-1267. (https://www.nature.com/articles/s41416-018-0063-4)

Patronek GJ, Waters DJ, Glickman 1997 Comparative longevity of pet dogs and humans: implications for gerontology research. J Gerontol A Biol Sci Med Sci 52(3):B171-178. https://www.ncbi.nlm.nih.gov/pubmed/9158552/

Pedersen JW, Gentry-Maharaj A, Fourkala EO, Dawnay A, Burnell M, Zaikin A, Pedersen AE, Jacobs I, Menon U, Wandall HH 2013 Early detection of cancer in the general population: a blinded case-control study of p53 autoantibodies in colorectal cancer. *British Journal of Cancer* **108**(1):107-114. (doi: 10.1038/bjc.2012.517.)

Poulos RC, Olivier J, Wong JWH 2017 The interaction between cytosine methylation and processes of DNA replication and repair shape the mutational landscape of cancer genomes. Nucleic Acids Res 45(13):7786-7795. (doi: 10.1093/nar/gkx463)

Rechavi O, Houri-Ze-evi L, Anava S, Goh WSS, Kerk SY, Hannon Gj Hobert O 2014 Starvation-induced transgenerational inheritance of small RNAs in C. elegans. Cell 158(2):277-287. doi:10.1016/j.cell2014.06.020

Sendowski K, Rajewsky MF 1991 DNA sequence dependence of gualnine-O6 alkylation by the N-nitroso carcinogens N-methyl- and N-ethyl-N-nitrosourea. *Mutation Research/Fundamentals and Molecular Mechanisms of Mutagenesis* **250**(1-2):153-160. (https://doi.org/10.1016/0027-5107(91)90171-J)

Sulak M, Fong L, Mika K, Chigurupati S, Yon L, Mongan N P, Emes RD, Lynch VJ 2016 TP53 copy number expansion is associated with the evolution of increased body size and an enhanced DNA damage response in elephants. *eLife* **5**:e11994. (https://www.ncbi.nlm.nih.gov/pubmed/27642012)

Tang J, Ning R, Zeng B, Li Y 2016 Molecular Evolution of PTEN pseudogenes in mammals. *PLoS One* **11**(12):e0167851. (https://www.ncbi.nlm.nih.gov/pmc/articles/PMC5148010/)

United States National Cancer Institute SEER Statistics Report, updated May 22, 2018. (https://www.cancer.gov/about-cancer/understanding/statistics)
