# Supplementary material for: Species-specific mechanisms of tumor suppression are fundamental drivers of vertebrate speciation: critical implications for the ‘war on cancer’
Source: Endocr Relat Cancer. 2018 Oct 12;26(2):C1–5. doi: 10.1530/ERC-18-0468 (PMC6347260; doi:10.1530/ERC-18-0468)
Supplement: Supplementary Section 2 [file supplementary_section_2.pdf]

Supplementary Section 2

Vitamin C auxotrophy is a critical component of the anthropoid primate-specific "kill switch" tumor suppression system

Although the focus of this supplement is the contribution of vitamin C auxotrophy to the stepwise evolution of the anthropoid primate-specific "kill switch" tumor suppression system, which reached its fullest expression in *Homo sapiens*, the necessity for this system derived from the advantage that increased body size and lifespan provided in the expansion of environmental niche beyond that of basal primates. We therefore must begin our discussion with a description of these body size and lifespan factors in primate evolution.

S2.1 Background: Primate speciation is characterized by stepwise increase in body size and lifespan

Modern primates are divided into two major clades, the more primeval Strepsirrhines (lemurs, logos, lorises) and the more recent Haplorrhines. Haplorrhine primates are further divided into anthropoid primates (monkeys, apes, humans) and tarsiers. Tarsiers are one of the smallest primates, weighing between 40-140 grams (1.4 - 4.9 ounces), and with a body length of 9 –16 cm (3.5 - 6.2 inches). They have possibly the slowest rate of development of any mammalian species, taking 6 months to attain a body weight of 23 grams. They are the only extant completely carnivorous primate, with their diet consisting of insects, lizards, and the occasional small bird. Tarsiers are placed in the Haplorrhine primate group because they have a dry rhinarium, and some repetitive DNA sequence elements that are similar to those of anthropoid primates (Hartig *et al*., 2013;

Schmitz *et al*. 2015). They have evolved very large eyes well suited for nocturnal hunting (albeit absent the reflective *tapetum lucidem* of nocturnal Strepsirrhine primates), and very long limbs designed for leaping at their prey. Basal Haplorrhine primates are thought to have resembled tarsiers, being similarly small, arboreal insectivores. Lending support to this idea, a complete skeleton of *Archicebus achilles,* a basal tarsier, was discovered in a bed of 55-million-year-old shale in China (Ni *et al*., 2013). *Archicebus* was even more diminutive than modern day tarsiers, just 7 cm (2.8 inches) long and weighing an estimated 20-30 grams (1 ounce). Detailed examination of this fossil revealed that the shape of the heel of *Archicebus* more resembled that of an anthropoid primate than a tarsier, suggesting that ancestral tarsiers and basal Haplorrhines were very similar.

The purpose of speciation is to expand the exploitation of environmental resources beyond those representing the niche of basal species (Supplementary Section 1). A frequent speciation strategy to expand beyond the environmental niche of basal species is to increase body size, which under natural circumstances generally leads to a parallel increase in lifespan as a matter of economy. Increases in body size can transform predator-prey relationships, enhance capacity for locomotion, enable access to new environmental resources that were unavailable to basal species, etc. This certainly appears to have been the case with Haplorrhine primates, which expanded in body mass from the few tens of grams of basal Haplorrhines and tarsiers, to the 220 kg (484 pounds) of the gorilla. Lifespan, too, underwent dramatic increases during primate evolution, with Strepsirrhine primates having lifespans of 12-15 years, compared to Haplorrhine primate lifespans of approximately 25 years. Any increase in

body size and lifespan beyond that of basal species requires species-specific mechanisms to suppress the amplified risk of malignant transformation that would otherwise be a consequence of such increases (Supplementary Section 1). As we shall now discuss, primate evolution is characterized by a very clear step-wise upgrading of species-specific tumor suppression mechanisms to enable the eight thousand-fold increase in body size from basal Haplorrhine primates to the largest members of this lineage.

S2.2 Primate-specific components of the kill switch tumor suppression system

We have previously described how the kill switch mechanism— irreversible uncompetitive inhibition of Glucose-6-phosphate Dehydrogenase (G6PD) leading to a catastrophic increase in intercellular ROS— requires high levels of circulating DHEAS, which only occur in primates (Figure 3 in Nyce, 2018). For uncompetitive inhibition of G6PD to reach irreversibility, accumulation of G6P substrate to high intracellular concentrations must also occur. This has been accomplished by selection for an anthropoid primate-specific sequence motif in the Glucose-6-phosphatase (G6PC) promoter (GAAT; Figure 2 in Nyce, 2018) that disables induction of G6PC activity, preventing catabolism of G6P to glucose and inorganic phosphate (P_i). Unlike anthropoid primates, tarsiers and Strepsirrhine primates retained the canonical GCAG G6PC promoter sequence motif that is characteristic of the vast majority of animal species. They therefore did not evolve the kill switch tumor suppression system, at least not to the extent that anthropoid primates did. Either their small size precluded necessity for the kill switch, or, more likely, their small size enabled a much less

optimized form of the kill switch to be sufficient, i.e., they required the evolution of only a rudimentary version of the kill switch.

S2.3 The inability to synthesize vitamin C is a further distinguishing feature of anthropoid primates

We now wish to discuss an additional lineage-specific trait of Haplorrhine primates that appears to be critically associated with the evolution of the adrenal androgen-mediated kill switch tumor suppression system; namely, loss of the ability to synthesize vitamin C (ascorbate). Most species, including Strepsirrhine primates, are capable of *de novo* synthesis of ascorbate (Smirnoff, 2018). However, loss of the capacity for *de novo* synthesis of vitamin C is a distinguishing feature of Haplorrhine primates, such that anthropoid primates (*ibid*.) and tarsiers (Pollock and Mullin, 1987), both have lost gulonolactone oxidase (GLO) activity, the final enzyme in ascorbate synthesis. Why?

The *de novo* synthesis of ascorbate, particularly in the presence of intracellular ROS, represents a significant sink for G6P in animals with GLO activity (Figure 1). Just as the catabolism of G6P to glucose and P_i *via* G6PC activity had to be disabled by conversion from the canonical GCAG to the GAAT sequence motif in the G6PC promoter of anthropoid primates to enable accumulation of G6P, so too, other sources of G6P loss had to be rendered inactive. Loss of GLO activity in Haplorrhine primates removed a pathway that would have consumed large amounts of G6P and would thereby have severely inhibited kill switch function. Thus, the accumulation of G6P required for the irreversible uncompetitive inhibition kinetics of DHEA toward G6PD, essential for the full expression of the anthropoid primate-specific kill switch tumor

suppression system, appears to require both the GAAT sequence motif which disabled catabolism of G6P by G6PC, and the loss of GLO activity. Anthropoid primates must therefore obtain ascorbate in their diets, which appears to have been an equitable tradeoff to enjoy optimization of the kill switch tumor suppression mechanism.

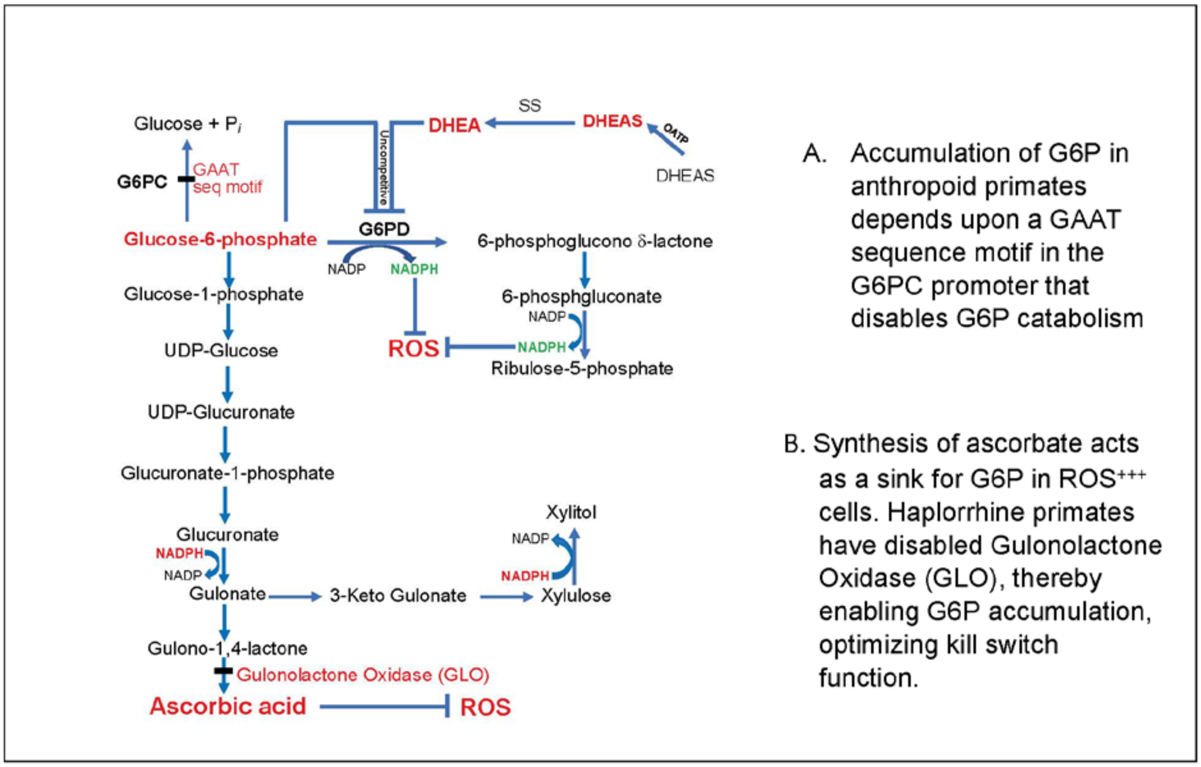

Figure 1. Synthesis of ascorbic acid acts as a sink for G6P in the presence of intracellular ROS. GLO activity is disabled in Haplorrhine primates, enabling accumulation of G6P in the presence of intracellular ROS. Both the GAAT sequence motif that disables DHEA-mediated induction of G6PC activity, and inactivation of GLO were required for optimization of the anthropoid primate-specific kill switch tumor suppression system. SS, Steroid Sulfatase; DHEAS (black type), extracellular DHEAS; DHEAS (red type), intracellular DHEAS.

S2.4 Step-wise evolution of the kill switch tumor suppression system enabled increased body size and lifespan of anthropoid primates

It is possible that GLO became superfluous in basal Haplorrhine primates and tarsiers because, like modern tarsiers, they were insectivorous and ingested sufficient vitamin C in their insect diet, insects representing a good source of vitamin C and most other nutrients (Finke, 2015). But as noted above, other possibilities also exist. We

discussed (Nyce, 2018, Supplementary Section 1 therein) the fact that both Haplorrhine and Strepsirrhine primates emerged in what is called the Paleocene Eocene Thermal Maximum (PETM), a period characterized by worldwide wild fires that caused an 8°C rise in the average world temperature, and consequent contamination of the worldwide landscape with polycyclic aromatic hydrocarbons (PAH). Basal Haplorrhines and tarsiers may already have had in place a basic form of the kill switch tumor suppression system, in which circulating DHEAS could be metabolized to DHEA in cells with PAH-induced p53 inactivation, and loss of GLO activity enabled some G6P to be accumulated, allowing rudimentary kill switch function. It is possible that such an elementary form of the kill switch, combined with their small body size, was sufficient to maintain cancer risk at the requisite 4% (Supplemental Section 1) in the PAH-contaminated landscape they inhabited. The anthropoid primates, however, had to increase their body size substantially to exploit new niches within the PAH-contaminated PETM. In order to increase their body size, improvements to the elementary form of the kill switch in basal primates and tarsiers were necessary. As noted above, one such major improvement was the conversion from the canonical GCAG sequence motif to GAAT, which disabled the catabolism of G6P by G6PC, enabling higher levels of intracellular G6P accumulation and thereby enhancing kill switch function. Another major improvement was a dramatic increase in the concentration of circulating DHEAS, to levels as much as forty-fold higher than in Strepsirrhine primates (Figure 3 in Nyce, 2018). These dramatic increases in circulating DHEAS trace the evolution toward greater body size and lifespan in Haplorrhine compared to Strepsirrhine primates. (The DHEAS levels in tarsiers remain unknown, as

they are a heavily protected endangered species. However, based upon their canonical GCAG G6PC promoter motif, we suspect that they will be in the range of Strepsirrhine, not Haplorrhine primates.) In any case, anthropoid primates diverged from tarsiers, increasing body size in ways that tarsiers (and Strepsirrhine primates) never did. The anthropoid primate-specific kill switch tumor suppressor system, characterized by extraordinarily high circulating DHEAS, the GAAT G6PC promoter sequence motif, and the inactivation of GLO, enabled this dramatic increase in body size and lifespan.

The picture that emerges is one in which circulating DHEAS, which opposes cortisol and thereby moderates the fight-or-flight response, may have originally appeared in primates to enable the formation of social groups much larger than would otherwise have been possible. Existing as a member of a large community would have significant survival value, with respect both to detecting and defending against predators, and providing more variability in the selection of mates. Subsequently, the Haplorrhine branch of the primate tree emerged with the deletion of GLO activity, activating a rudimentary form of the kill switch that may have enabled exploitation of PAH-contaminated resources within the fire-ravaged landscapes of the PETM. Then, in order to expand niche exploitation by increasing body size and lifespan, anthropoid primates emerged with their GAAT sequence motif in the G6PC promoter, improving kill switch function by optimizing G6P accumulation above that enabled by GLO deletion. Finally, selection for increased levels of circulating DHEAS occurred, culminating in humans, the only anthropoid primate to harness fire (Figure 2). The harnessing of fire may not have been possible without the kill switch tumor suppression mechanism already being present at an early time in hominin evolution. The species-specific PAH exposure that

would have occurred by consuming heat processed food, and by smoke inhalation that was unavoidable in the unventilated habitats of early *Homo* species, would likely have selected for higher and higher levels of circulating DHEAS, accounting for the fact that *Homo sapiens* have by far the highest levels of any anthropoid species.

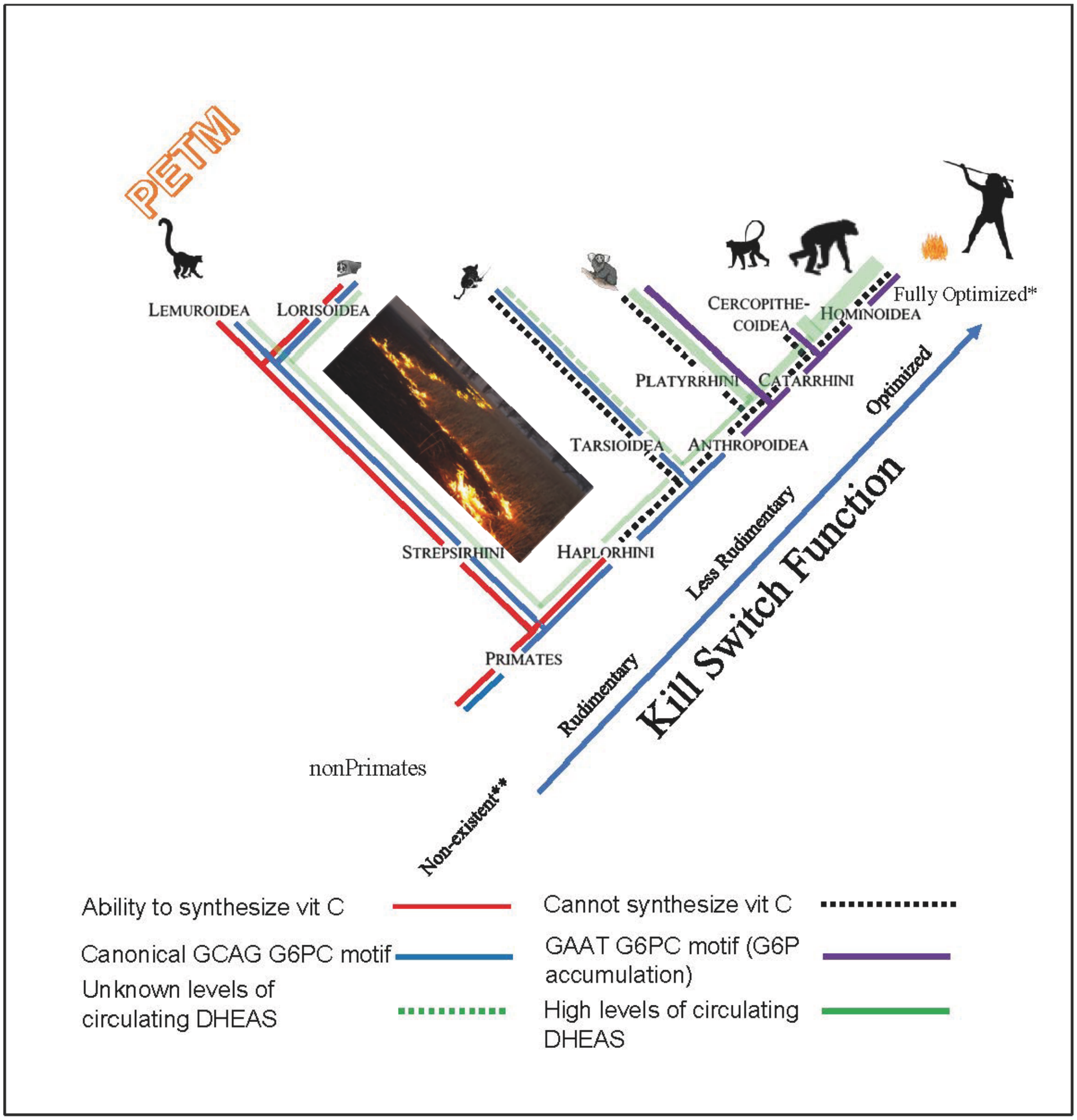

Figure 2

S2.5 Conclusion

It cannot be coincidental that three major, primate-specific evolutionary events—circulating DHEAS, inactivation of GLO resulting in inability to synthesize vitamin C, and the GAAT sequence motif of G6PC— have a common intersection in the enabling of irreversible uncompetitive inhibition of G6PD which requires high concentrations of substrate and inhibitor; DHEAS by acting as a safe, circulating form of the uncompetitive inhibitor, DHEA; and GLO inactivation and the GAAT G6PC sequence motif combining to enable accumulation of G6P substrate. Determining if reconstitution of circulating DHEAS will restore the kill switch tumor suppression system, and if such restoration will normalize human lifetime cancer risk to the 4% of other long-lived vertebrate species (Abegglen *et al*., 2015), is an experiment that can only be performed in humans. But unlike DHEA, DHEAS should be a safe pharmacological test substance. In view of this fact, and the dramatic increases in worldwide cancer cases predicted for the near future, we recommend that consideration be given to including the cost of such clinical trials in the National Cancer Institute's 2020 budget, which has just been opened for consideration.

Acknowledgements. Very helpful comments on aspects of the manuscript that touched on primate evolution were provided by Dr. Juergen Schmitz of the Institute of Experimental Pathology (ZMBE), University of Muenster; his assistance is gratefully acknowledged.

References

Abegglen LM, Caulin AF, Chan A, Lee K, Robinson R, Campbell MS, Kiso WK, Schmitt DL, Waddell PJ, Bhaskara S, *et al*. 2015 Potential mechanisms for cancer resistance in elephants and comparative cellular response to DNA damage in humans. *JAMA* **314** 1850-1860. (https://doi.org/10.1001/jama.2015.13134)

Berg JM, Tymoczko JL, Stryer L 2002 The glycolytic pathway is tightly controlled. In Biochemistry, 5th Edition, section 16.2. WH Freeman, New York. (https://www.ncbi.nlm.nih.gov/books/NBK22395/)

Campbell BC 2006 Adrenarche and the evolution of human life history. *American Journal of Human Biology* **18**(5):569-589. (DOI: 10.1002/ajhb.20528)

Finke MD 2015 Complete nutrient content of four species of commercially available feeder insects fed enhanced diets during growth. *Zoo Biology* **34**:554-564. (https://onlinelibrary.wiley.com/doi/pdf/10.1002/zoo.21246)

Hartig G, Churakov G, Warren WC, Brosius J, Makalowski W, Schmitz J 2013 Retrophylogenomics place tarsiers on the evolutionary branch of anthropoids. *Scientific Reports* 3:1-6. (DOI: 10.1038/srep01756)

Nguyen TV, McCracken JT, Ducharme S, Cropp BF, Botterton KN, Evans AC, Karama S 2013 Interactive effects of dehydroepiandrosterone and testosterone on cortical thickness during early brain development. *J Neuroscience* **33**(26):10840-8. (Doi: 10.1523/jneuroscience.5747-12.2013)

Nguyen TV, Wu M, Lew J, Albaugh MD, Botterton KN, Hudziak JJ, Fonov VS, Collins DL, Campbell BC, Booij L, *et al*. 2017 Dehydroepiandrosterone impacts working

memory by shaping cortico-hippocampal structural covariance during development. *Psychoneuroendocrinology* **86**:110-121. (doi: 10.1016/j.psyneuen.2017.09.013.)

Ni X, Gebo DL, Dagosto M, Meng J, Tafforeau P., Flynn JJ, Beard KC 2013 The oldest known primate skeleton and early haplorrhine evolution. *Nature* **498**:60-64. (https://www.nature.com/articles/nature12200)

Nyce JW 2018 Detection of a novel, primate-specific "kill switch" tumor suppression mechanism that may fundamentally control cancer risk in humans: An unexpected twist in the basic biology of TP53. Endocrine-Related Cancer 25(11):R497-517. https://www.ncbi.nlm.nih.gov/pmc/articles/PMC6106910/pdf/erc-25-R497.pdf

Pollock JI, Mullin RJ 1987 Vitamin C biosynthesis in prosimians: Evidence for the anthropoid affinity of Tarsius. *American Journal of Physical Anthropology* **73**(1):65-70. (https://doi.org/10.1002/ajpa.1330730106)

Quinn T, Greaves R, Badoer E, Walker D 2018 DHEA in prenatal and postnatal life: Implications for brain and behavior. *Vitam Horm* **108**:145-174. (doi: 10.1016/bs.vh.2018.03.001.)

Schmitz J, Noll A, Raabe CA, Churakov G, Voss R, Kiefmann M, Rozhdestvensky T, Brosius J, Baertsch R, Clawson H *et al*. 2015 Genome sequence of the basal haplorrhine primate Tarsius syrichta reveals unusual insertions. *Nature Communications* **7**:1-11. (DOI: 10.1038/ncomms12997)

Smirnoff N 2018 Ascorbic acid metabolism and functions: A comparison of plants and mammals. *Free Radical Biology and Medicine* **122**:116-129. (https://www.sciencedirect.com/science/article/pii/S0891584918301369?via%3Dihub#f0005)
